# Miscellaneous

**Published:** 1893-04

**Authors:** 


### MISCELLANEOUS.

### SOFT AND HARD WATER.

Everybody likes soft water, but many half-scientific people have a kind of idea that hard water—that is, water with carbonate of lime dissolved in it—may be of some value in the nutrition and development of bones, and especially in the development of children's bones. "Nothing of the kind," says in effect Dr. J. M. Fox, in a paper addressed to the Sandbach Local Board of Health. The principal use of water in the human body is for solvent purposes, argues Dr. Fox. But if that be so, it is manifest that water which has 70, or 80, or even 100 grains of solid matter per gallon dissolved in it must be much less powerfully solvent than water which has not more than five or ten grains. The water which is used up in dissolving the lime cannot dissolve other soluble substances—at any rate, not to the full extent of the natural solvent power of unadulterated water.

It is sometimes argued, as we have said, that water having lime dissolved in it may, when drunk, give up its lime to the body, and so help in the formation of bones. On this point Dr. Fox quotes Sir Lyon, now Lord Playfair. On being questioned by the Royal Commission, he replied as follows: "I have seen evidence given in cases of water supply, not only that it was desirable for health, but that it (carbonate of lime) was absolutely necessary for the formation of bones, But that showed a lamentable want of chemical knowledge, because the lime required in food does not come from the water, but from the solid particles of food taken, and I do not think that the lime in water has any influence on the process of animal nutrition." Sir Lyon went on to add that "the water consumed in the mountainous districts of Scotland is soft water, and Highlanders are not generally supposed to be deficient in bone or muscle." On this same point, Dr. Angus Smith, formerly Chief Inspector of Alkali Works, stated before the same Royal Commission that he thought "that the tallest people in Great Britain are to be met with in soft-water districts, for instance, in Cumberland, and probably Aberdeen." The tallest people of all were found in Aberdeen, "which is a very soft-water district." Soft-water, is, in short, pure water, so far as lime is concerned; and both in sickness and in health, and, indeed, for all the ordinary purposes for which water is required, it is much to be preferred.—*Hospital.*

---

A Child Turning to Stone.—The French Academy of Sciences has been making reports on an extraordinary case of selerema or petrifying of the skin and outer tissues of a human body. The case under consideration, which, by the way, is one of the rarest reported in medical literature, is that of an eighteen months old child of St. Jeanne, a suburb of the French metropolis. When this doomed child was last made the subject of a clinic its flesh was cold and almost as hard as marble; and, while it still continues to live, it can only move the eyelids and lips. The poor little sufferer sleeps nearly all the time, lying with its eyes wide open and breathing more like some cleverly devised automaton than a human being. The inner side of the lips, that portion of the eyelids which folds up under the eyebrows, and a place about the size of a silver dollar under each arm, are the only spots on the body which present any of the warmth or pliability characteristic of human flesh. In June or July the child was as healthy as any of St. Jeanne's many babies, until it got a heavy fall, striking on the back of the head. The disease, which dates from this fall, and seems to have some mysterious connection between the tissue and the skin, is supposed to be the result of the nervous shock. According to my data, this is the thirty-ninth case on record and the second in which the whole of the body was affected. The doctors in attendance say that death is the only relief.

Cutting Children's Hair —As a general rule, to which, however, there are occasional exceptions, I believe that nature intends boys' hair to be cut short, and girls' hair to be left long. Advice in accordance with this theory seems to have been given more than eighteen hundred years ago to the Corinthians. Here is an example to support the general rule. A very little girl, who was, and still is, remarkable for the luxuriance of her hair, got hold of a pair of scissors and sheared off a large tress from over her forehead. Whereat her mother wept, for she said that the gap would never be properly filled up again. And the mother was right ; for though many years have elapsed, a gap over the brow is still discernible amid the wealth of hair that now grows from that girl's head. But here is one of the exceptions. A little girl had thin, scanty hair, and under good advice it was cut quite short. Now, after many years, it is still somewhat short and slow in growing, but it has improved in strength and thickness very satisfactorily. I should advise letting good hair alone ; but if a girl's hair be weak or thin, or slow in growing, I would risk cutting it short, and let the girl have the head of a boy for a while. It may also be treated with some such thing as brilliantine.

A Well-dressed Woman.—Some women will give a certain air of style, and even elegence, to a simple toilette, while others look dowdy and commonplace in costly, but ill-assorted clothes. Materials should always be of as good quality as the means will permit, but if you cannot afford both, better have a cheaper material well made than a richer quality made by some one who does not really understand the first principles of the art of dressmaking. The woman who always looks well-dressed is the one who chooses her toilettes with good taste, and with reference to other articles of her wardrobe. As a rule, she will choose quiet colors, knowing that they are not only the most economical, but the prettiest, for nearly if not all occasions. For this reason black is a favorite color with persons of good taste and limited means, but we cannot always go in such a sombre garb, however elegant, and spring and summer call imperatively for lighter ones ; they need not necessarily be of blue, pink or other bright hues, which are conspicuous in the extreme for street wear, as there are many others, all shades of tan, grey, and pale mauve, which make up exceedingly well.

Alcoholic Poisoning.—Two cases of alcoholic poisoning are said to have occurred at St. Helens recently. One was that of a youth fifteen years of age, who was sent by his employers with some spirits in a stone jar to a customer. He drew the cork, and is supposed to have drank about 10oz. ; he became comatose, and died next morning. Last week a painful case, which was also fatal, occurred at Wigan. A young, healty man, laboring in a railway goods yard, drank from a can a quantity (said to be from 10 to 15 oz.) of whisky, which had been extracted from puncheons that were being returned as empties. In a very short time afterwards he was found lying on the floor, and was left there to sleep the effects of the alcohol off till 7 p.m., when Mr. Berry was called, who found him insensible and almost in a dying condition. All efforts to restore him to consciousness failed, and he died early next morning. A post-mortem examination proved conclusively that death occurred from alcoholic poisoning.

Saved by a Dog.—It is related that Mrs. Robert Lynn, of Marshall, Ill., was awakened on the night of February 16th, by her large Newfoundland dog rubbing his cold nose on her face and howling. She awoke to find the house on fire and her husband partly suffocated. By great effort they both escaped being burned to death. The fatihful dog, who had thus undoubtedly saved them from an awful fate, immediately on being released went bounding across fences to the home of a brother of Mrs. Lynn, who lived a square away. Meeting him and his wife on the way, they having been roused by the cries of fire, he bounded upon them and then darted back toward the burning house, looking back at them and barking impatiently. Then he would rush back to them, and, seizing hold of their clothing, strive to urge them along faster. A human being could not have displayed more apparent reasoning power than this faithful dog.

Curious Facts about the Pump.—The common water pump of to-day is but an improvement on a Grecian invention which first came into general use during the reign of the Ptolemies, Philadelphos, and Energetes, 283 to 221 B. C. The name which is very similar in all languages, is derived from the Greek word "Pempo," to send or throw. The most ancient description we have of the water pump is by Hero of Alexander. There is no authentic account of its general use outside of Egypt previous to its introduction into the German provinces at about the opening of the Sixteenth Century. Pumps with plungers and pistons were invented by Moreland, an Englishman, in 1674; the double-acting pump by De la Hire, the French academician, some twenty years later.

Nutritive Value of Potatoes.—A German chemist has recently been investigating the nutritive value of potatoes, and finds that it varies considerably according to the age of the potato. The amount of starch that the potato contains varies from month to month, as follows, beginning with the tuber when not yet fully developed: August, 10 per cent.; September, 14 per cent.; October and November, 15 per cent.; December, 17 per cent.; January, 17 per cent.; February, 16 per cent.; March, 15 per cent.; April, 12 per cent.; May 10 per cent. Potatoes are therefore most nutritious in the winter, when their food value is of greatest service.

New Way of Relieving Hiccough.—Hiccough is due to the spasmodic contraction of the diaphragm. This is the result of the irritation of the ends of the phrenic and pneumogastric nerves in the stomach acting reflexly upon the diaphragm. The exciting condition of the nerves can be overcome by a simple pressure of the index finger, just above the upper end of the sternum.

Cicero.—Some of Cicero's sentiments are noble and touching, as this: "I do not despise life, nor do I regret my own existence, since I have so lived that I think I can truly say I was not born in vain; and I shall depart this life not as one who leaves home, but as one who sets out from a tavern by the roadside."

Advice to Mothers.—Are you disturbed at night and broken of your rest by a sick child suffering and crying with pain of cutting teeth? If so, send at once and get a bottle of "Mrs. Winslow's Soothing Syrup," for children teething. It will relieve the poor little sufferer immediately. It cures Dysentery and Diarrhœa, regulates the Stomach and Bowels, cures Wind Colic, softens the Gums, reduces Inflammation, and gives tone and energy to the whole system. Be sure and ask for "Mrs. Winslow's Soothing Syrup," and take no other kind.

The Royal Baking Powder still holds the front rank in the estimation of housewives and cooks. Those who have used it once will usually have no other. Sold by all grocers.

